# Role of IL-16 in CD4^+^ T cell-mediated regulation of relapsing multiple sclerosis

**DOI:** 10.1186/s12974-015-0292-x

**Published:** 2015-04-22

**Authors:** Dusanka S Skundric, William W Cruikshank, Jelena Drulovic

**Affiliations:** Department of Immunology and Microbiology, Wayne State University, 540 East Canfield Av., Detroit, MI USA; Pulmonary Center Boston University, 715 Albany Street, Boston, MA USA; Clinic of Neurology, Clinical Center of Serbia, Faculty of Medicine, University of Belgrade, Dr Subotica 6, Belgrade, Serbia

**Keywords:** IL-16, Multiple sclerosis, Cerebellum, CD4+ T cells, IFN-β, FoxA1, MOG, EAE, Translational, Therapy

## Abstract

In an important article published in Nature Medicine, Liu and colleagues described a novel CD4^+^ FoxA1^+^ regulatory T (Treg) cell population as distinct regulators of relapsing-remitting multiple sclerosis (RRMS) and experimental autoimmune encephalomyelitis (EAE). CD4^+^ FoxA1^+^ Treg cells appear as key regulators of responsiveness to therapy with interferon beta (IFN-β) in RRMS patients. Data indicate that CD4^+^FoxA1^+^ FOXP3^−^ Treg cells develop within the central nervous system (CNS), and a potential of cerebellar granule neurons (CGN) in generation of CD4^+^FoxA1^+^PD-L1^hi^FOXP3^−^ Treg cells from encephalitogenic CD4^+^ T cells.

A CD4 co-receptor specific ligand, IL-16, governs trafficking and biological properties of CD4^+^ T cells irrespective of their activation state. Functions of IL-16, relevant to Treg cells, include expansion of CD4^+^CD25^+^ T cells in long-term cultures with IL-2, *de novo* induction of FOXP-3 and migration of FOXP-3^+^ T cells. IL-16 is highly conserved across species including human and mouse. CGN and neurons in hippocampus contain neuronal-IL-16 (NIL-16), splice variant of immune IL-16, and express CD4 molecule. In a CD4-dependent manner, IL-16 supports cultured CGN survival.

Concomitant studies of RRMS lesions and corresponding MOG_35–55_-induced relapsing EAE in (B6 × SJL)F1 (H-2^b/s^) mice discovered similar roles of IL-16 in regulation of relapsing disease. In RRMS and EAE relapse, peak levels of IL-16 and active caspase-3 correlated with CD4^+^ T cell infiltration and levels of T-bet, Stat-1(Tyr^701^), and phosphorylated neurofilaments of axonal cytoskeleton [NF (M + H) P], suggesting a role of locally produced IL-16 in regulation of CD4^+^ Th1 inflammation and axonal damage, respectively. IL-16 was abundantly present in CD4^+^ T cells, followed by CD20^+^ B, CD8^+^ T, CD83^+^ dendritic cells, and Mac-1^+^ microglia. Apart from lesions, bioactive IL-16 was located in normal-appearing white matter (NAWM) and normal-appearing grey matter (NAGM) in RRMS brain and spinal cord.

A cytokine IL-16 emerges as an important regulator of relapsing MS and EAE. Better understanding of immune cell-neuron interactions mediated by IL-16 will foster development of more specific CD4^+^ T cell subset-targeted therapies to prevent or ameliorate progression of neuroinflammation and axonal and neuronal damage. Translational studies necessitate corresponding EAE models.

## Background

Discovery of a novel lineage of regulatory T (Treg) cells, which are CD4^+^FoxA1^+^, as major regulators of responsiveness to IFN-β therapy in patients with relapsing-remitting multiple sclerosis (RRMS), is important for further optimization of this first in class disease-modifying therapy (DMT). Concurrent clinical and experimental evidence support the notion that regulation of MS progression engages distinct regulatory pathway carried by CD4^+^FoxA1^+^ FOXP3^−^ Treg cells, which develop within the central nervous system (CNS) [1]. To gain better understanding of complex cellular and molecular interactions important for disease progression, some fundamental mechanisms underlying MS immune pathogenesis, reinforced by this recent finding deserve additional attention and reappraisal.

Primarily, a potential of locally inflamed CNS microenvironment to regulate plasticity of CD4^+^ T cell responses, by switching their functional phenotypes from encephalitogenic to suppressive, regulatory, FoxA1 mediated, appears vital for progression of the disease [1]. Locally elaborated cytokines are critical regulators of types of inflammatory cell interactions and mediators of tissue damage. In progressive inflammatory disease, where inflammatory mediators are reoccurring, immune cell communications are likely diverse engaging in distinctive types of relationships throughout RRMS and its conversion into secondary progressive (SP) disease.

Additionally, effects of IFN-β therapy regulated through CD4^+^ FoxA1^+^ Treg are shared mechanisms between RRMS and EAE [1]. Such approach further emphasizes importance of an appropriate EAE model when studying immune regulation of MS. In order to identify ongoing modifications in intertwined shared regulatory mechanisms, it is critical for an EAE model to resemble the RRMS clinical course, histopathology and relevant immune mechanisms [2,3]. Immune regulation of relapse in response to myelin oligodendrocyte glycoprotein peptide 35 to 55 (MOG_35–55_) is poorly studied due to a relative deficiency of relapsing EAE in mice [4]. MOG is conserved across species. An immunodominant epitope of MOG_35–55_ is associated with autoimmune responses by CD4^+^ Th1 and Th17 cells and/or autoantibodies in RRMS and aquaporin-4^−^ (AQP4^−^) relapsing neuromyelitis optica (NMO), respectively [5].

Finally, data from co-culture experiments prompt us to question the nature of interactions between cerebellar granule neurons and encephalitogenic CD4^+^ T cells, in generation of CD4^+^FoxA1^+^PD-L1^hi^FOXP3^−^ Tregs [1]. Demyelinating lesions in cerebellar cortex are prominent in progressive MS [6]. Clinical signs of cerebellar dysfunction including nystagmus and ataxic gate accompany MS [7]. In homeostasis, hippocampal and cerebellar granule neurons produce neuronal-IL-16 (NIL-16), a splice variant of immune IL-16, which is produced by lymphocytes, macrophages, DC, mast cells, and microglia [8-10].

## Commentary

Data from RRMS tissue analysis and relapsing EAE are concurrent in identifying a CD4^+^ T cell-specific chemotactic factor, IL-16, as a key regulator of progressive CNS inflammation mediated by CD4^+^ Th1 cells.

In RRMS acute brain lesions, IL-16 was found in CD3^+^ and CD4^+^ T cells, followed by CD20^+^ B, CD8^+^ T and CD83^+^ dendritic cells and Mac-1^+^ microglia. Often, IL-16 located to cell cytoplasm, less frequently to cell nucleus, or polarized on the membrane connecting adjoining cells. The majority of CD4^+^ IL-16^+^ cells were T-bet^+^ and active caspase-3^+^. Increased levels of bioactive IL-16 correlated with levels of active caspase-3, T-bet, Stat-1 (Tyr^701^), and phosphorylated medium and heavy neurofilaments of axonal cytoskeleton, [NF (M + H) P], which suggested a role for IL-16 in regulation of CD4^+^ Th1 inflammation and damage of axonal cytoskeleton, respectively. IL-16 induces migration of CD4^+^Tbet^+^ (Th1) and CD4^+^FOXP3^+^ (Treg) cells and FOXP3 *de novo* expression [11,12]. In the MS brain, highest levels of IL-16 were detected in chronic lesions, followed by subacute and acute. Conversely, in MS spinal cord, acute lesions contained highest levels of IL-16. Bioactive IL-16 was also detected in normal-appearing white matter (NAWM) and normal-appearing grey matter (NAGM) in the brain and NAWM in the spinal cord. More robust increase of IL-16 was noted in the spinal cord compared to brain NAWM. A sharp increase of pro-IL-16 was found in NAGM in RRMS brain [2]. Data propose regional differences in IL-16 regulation in lesions between the brain and spinal cord and a role of bioactive and pro-IL-16 in pathology of NAWM and NAGM in RRMS.

Corresponding to our findings of elevated IL-16 levels *in situ*, raised levels of IL-16 were detected in serum of MS patients. Following therapy with IFN-β1a, IL-16 gene expression in peripheral blood mononuclear cells (PBMC) and its serum levels were down regulated, suggesting IL-16 as a potential biomarker for MS [13,14]. Myelin oligodendrocyte glycoprotein (MOG)-specific CD4^+^Th17 exhibited more pronounced IL-16 production compared to CD4^+^Th1 cells, implicating a role of IL-16 in the regulation of Th17 responses [14].

Concurrent with RRMS findings, in relapsing-remitting MOG_35–55_-induced EAE in (B6 × SJL) F1 (H-2^b/s^) mice, intra-CNS production of IL-16 correlates with extensive CD4^+^ T cell infiltration, accompanied by phosphorylation of axonal cytoskeleton, which all peak during relapses and in chronic progressive disease. IL-16 and CD4 were co-precipitated from CNS of relapsing H-2^b/s^ mice. Data indicate that active caspase-3-mediated release of bioactive IL-16 from infiltrating CD4^+^ T cells supports progression of local inflammation by specifically enhancing trafficking of additional CD4^+^ T cells [2,3,15].

As opposed to low/non-relapsing parental, B6 (H-2^b^), in H-2^b/s^ mice, immunization with MOG_35–55_ induces relapsing-remitting EAE with extensive CD4^+^ T cell infiltration, followed by Mac-3^+^ macrophages, B220^+^ B, and CD8^+^ T cells. Induced autoimmune responses to MOG_35–55_ in H-2^b/s^ but not in H-2^b^ mice result in myelin-associated glycoprotein (MAG) predominated pattern of demyelination, which is suggestive of oligodendrocyte dysfunction and/or damage [15,16]. A similar pattern of demyelination was observed in the MS lesion subtype III, where profound loss of MAG serves as an indicator of a distal oligodendrogliopathy [17]. MAG-predominated demyelination, a decrease of NF160 axonal neurofilament, and a sharp elevation of PARPp85 suggested axonal dysfunction and irreversible apoptosis, respectively, and correlated with severe relapsing disease in H-2^b/s^ mice [16]. Relapsing-remitting EAE in H-2^b/s^ mice may provide valuable insights into mechanisms of MAG depletion and oligodendroglial pathology initiated by autoimmune responses to MOG_35–55_. Therapy with IL-16-specific antibody diminished paralysis and CD4^+^ T infiltration and abrogated demyelination and axonal damage in relapsing EAE H-2^b/s^ mice [18].

Studies from RRMS and corresponding MOG_35–55_-induced relapsing EAE in H-2^b/s^ mice suggest a role of IL-16 in neuron-immune cell communications in lesions of gray matter, including cerebellar and hippocampal, and in adjacent NAGM and NAWM (Skundric, unpublished). Cortical and subcortical, including hippocampal, grey matter lesions underlie cognitive deficits observed in MS [19].

Beneficial effects of IFN-β therapy include potential lowering of probability of disease progression from RRMS to SP and development of Expanded Disability Status Scale (EDSS) score [20].

Uncovering those IL-16-specific mechanisms is critical for the development of new CD4^+^ T cell subset-targeted therapies for MS and other chronic and/or progressive inflammatory and demyelinating diseases.

## Conclusion

Data from FoxA1 co-culture experiments argue for the importance of bidirectional neuron-immune cell communications and underscore necessity for better understanding of underlying mechanisms, in particular IL-16 mediated. Future studies in this direction are required for the development of specifically targeted therapies aimed at preventing or ameliorating neuronal damage.

Delineating differences in mechanisms of immune regulation between the spinal cord and brain is critical for therapy of NMO and NMO-spectrum disorders (NMOSD) with predominate optic nerve and spinal cord demyelination.

Delineating molecular mechanisms enhancing migration of encephalitogenic CD4^+^ cells (Th1 and Th17), and Treg cells into the CNS throughout the disease, is essential in the development of new therapy specific for CD4^+^ T cell subsets for MS and similar inflammatory and demyelinating diseases.

## Abbreviations

[NF(M + H)]P: phosphorylated medium and heavy chains of neurofilament
AQP4: aquaporin-4
DMT: disease-modifying therapy
EAE: experimental autoimmune encephalomyelitis
IFN-β: interferon beta
IL-16: interleukin 16
MAG: myelin-associated glycoprotein
MOG: myelin oligodendrocyte glycoprotein
NAGM: normal-appearing grey matter
NAWM: normal-appearing white matter
NIL-16: neuronal IL-16
NMO: neuromyelitis optica
NMOSD: NMO-spectrum disorders
PBMC: peripheral blood mononuclear cells
RRMS: relapsing-remitting multiple sclerosis
Stat-1: signal transducer and activator of transcription-1
